# Raw Pig Blood Consumption and Potential Risk for *Streptococcus suis* Infection, Vietnam

**DOI:** 10.3201/eid2011.140915

**Published:** 2014-11

**Authors:** Vu Thi Lan Huong, Ngo Thi Hoa, Peter Horby, Juliet E. Bryant, Nguyen Van Kinh, Tran Khanh Toan, Heiman F.L. Wertheim

**Affiliations:** Oxford University Clinical Research Unit, Hanoi, Vietnam (V.T.L. Huong, P. Horby, J.E. Bryant, H.F.L. Wertheim); Oxford University Clinical Research Unit, Ho Chi Minh City, Vietnam (N.T. Hoa);; University of Oxford, Oxford, UK (V.T.L. Huong, N.T. Hoa, P. Horby, J.E. Bryant, H.F.L. Wertheim);; National Hospital for Tropical Diseases, Hanoi (N.V. Kinh);; Hanoi Medical University, Hanoi (T.K. Toan)

**Keywords:** raw pig blood, consumption, practices, perceptions, zoonoses, risk factor, Streptococcus suis, bacteria, Vietnam

## Abstract

We assessed consumption of raw pig blood, which is a risk factor for *Streptococcus suis* infection in Vietnam, by using a mix-method design. Factors associated with consumption included rural residency, age, sex, occupation, income, and marital status. We identified risk groups and practices and perceptions that should be targeted by communication programs.

Consumption of undercooked animal products is a well-established risk factor for acquiring many infectious diseases (*1**–**5*). In Vietnam, raw blood of pigs or other animals is consumed in a dish known as *tiet canh*. The main ingredients of porcine *tiet canh* include coagulated, fresh, uncooked blood mixed with chopped cooked pork tissues (Figure). A recipe is shown in Technical Appendix Table 1. Consumption of raw pig products is associated with trichinellosis and *Streptococcus suis* meningitis in humans in Vietnam (*6**–**8*).

**Figure:**
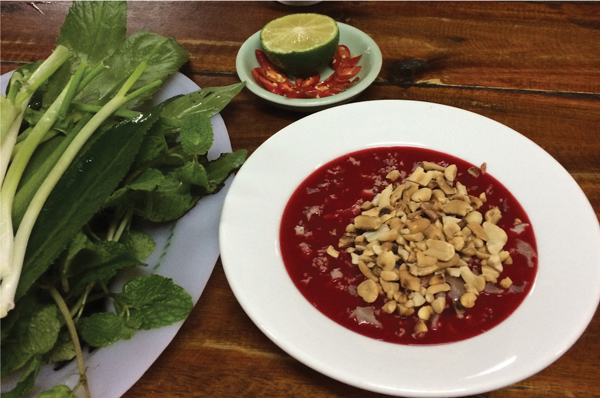
Traditional dish (*tiet canh*) containing raw pig blood, Vietnam.

*S. suis* is a common gram-positive bacterium found in pigs, which can cause severe infections in humans; ≈90% of human cases are reported from Asia (*9**,**10*). Case-fatality rates range from 3% to 7% but may reach ≈60% among patients with severe sepsis, as observed in a large outbreak in Sichuan, China, in 2005 (*11*). Studies have identified occupational exposure to pigs and consumption of specific traditional pork dishes as key risk factors for contracting *S. suis* infection (*10*). Effective control of diseases transmitted through consumption of undercooked pig products requires a thorough understanding of this food practice. Therefore, we investigated consumption of porcine *tiet canh* in northern Vietnam and explored community perceptions regarding associated disease risks.

## The Study

The study was conducted in 2 health care and demographic surveillance sites in Hanoi Province, Vietnam: Ba Vi District (rural) and Dong Da District (urban). Each site contained ≈11,000 households that were selected by cluster sampling to represent the district population (*12**)*. This study was approved by ethical committees at the University of Oxford and Hanoi Medical University.

A quantitative survey on *tiet canh* consumption was administered to household members at health care and demographic surveillance sites (Ba Vi: May–June 2012; Dong Da: December 2012–January 2013). Field surveyors visited households as part of their routine survey schedules and interviewed 1 member per household individually. A total of 6,993 participants in Ba Vi and 3,991 participants in Dong Da were interviewed (no households refused). After persons for whom no data were available regarding age and sex were excluded, 6,943 (99.3%) persons in Ba Vi and 3,921 (98.2%) in Dong Da were included in the analysis (mean age [range]: 47.0 [8–97] years in Ba Vi and 48.3 [9–102] years in Dong Da).

Rural and urban respondents differed significantly by sex (24.6% vs. 34.5% male participants, respectively), education (21.9% vs. 74.3% with ≥10 years of education), and occupation (2.4% vs. 29.6% office workers). Subsequently, 10 focus groups that involved 81 participants in the 2 districts were formed (April–June 2013). Participants in focus groups were selected on the basis of reported consumption of *tiet canh* in the previous survey and were stratified by district, sex, and consumption status. For each district, 1 focus group was also conducted for local government workers. Details on data collection, characteristics of participants, and data analysis are described in the Technical Appendix.

A total of 35% (95% CI 33.8%–36.1%) of persons in the rural area vs. 8.6% (95% CI 7.7%–9.5%) in the urban area reported eating porcine *tiet canh* in the past year. Duck blood was the second most common source of *tiet canh* (Technical Appendix Table 3). Subsequent analyses were restricted to porcine *tiet canh*. Sex, age, level of education, occupation, economic status, and marital status were associated with consumption patterns by univariate analysis (Table 1). However, level of education was not associated by multivariable regression (Table 2).

**Table 1:** Factors associated with consumption of raw pig blood among respondents in 2 districts of Hanoi, Vietnam*

Factor	Ba Vi District (rural)			Dong Da District (urban)		
	Consumption, no. (%)	No consumption, no. (%)	OR (95% CI)	Consumption, no. (%)	No consumption, no.(%)	OR (95% CI)
Sex						
M	900 (52.6)	810 (47.4)	**3.0 (2.7–3.4)**	250 (18.5)	1,103 (81.5)	**6.4 (4.9–8.2)**
F	1,527 (29.2)	3,706 (70.3)	1	88 (3.4)	2,480 (96.6)	1
Age, y						
<20	17 (13.8)	106 (86.2)	**0.4 (0.2–0.7)**	2 (4.1)	47 (95.9)	0.8 (0.2–3.7)
20–29	209 (28.8)	516 (71.2)	**1.5 (1.2–1.8)**	37 (8.3)	411 (91.7)	**1.7 (1.1–2.7)**
30–39	535 (38.3)	863 (61.7)	**2.3 (2.0–2.8)**	68 (8.7)	713 (91.3)	**2.2 (1.5–3.4)**
40–49	759 (42.3)	1,037 (57.7)	**2.6 (2.2–3.0)**	85 (11.3)	668 (88.7)	**2.8 (1.9–4.1)**
50–59	593 (36.2)	1,046 (63.8)	**1.9 (1.6–2.2)**	104 (11.2)	823 (88.8)	**2.6 (1.8–3.8)**
≥60	314 (24.9)	948 (75.1)	1	42 (4.4)	921 (95.6)	1
Education, y						
≤5	303 (27.3)	807 (72.7)	1.5 (1.0–2.1)	10 (6.8)	138 (93.2)	2.2 (1.0–4.5)
6–9	1,637 (38.0)	2,673 (62.0)	**1.7 (1.2–2.5)**	97 (11.4)	755 (88.6)	**2.0 (1.5–2.8)**
10–12	441 (32.6)	910 (67.4)	1.3 (0.9–1.9)	124 (7.9)	1,450 (92.1)	1.1 (0.9–1.5)
>12	44 (26.7)	121 (73.3)	1	103 (7.8)	1,218 (92.2)	1
Occupation						
Office worker	41 (24.4)	127 (75.6)	1	88 (7.6)	1,067 (92.4)	1
Manual laborer†	277 (41.6)	389 (58.4)	**1.5 (1.0–2.3)**	55 (16.2)	284 (83.8)	**1.9 (1.3–2.8)**
Services and sales	195 (34.8)	366 (65.2)	**1.6 (1.0–2.3)**	113 (12.2)	810 (87.8)	**1.9 (1.4–2.5)**
Farmer	1,649 (36.9)	2,825 (63.1)	**2.0 (1.4–2.9)**	0	2 (100)	-
Other	98 (37.7)	162 (62.3)	1.4 (0.9–2.3)	12 (21.4)	44 (78.6)	2.1 (1.1–4.3)
Not working‡	156 (19.8)	630 (80.2)	1.1 (0.7–1.6)	69 (4.8)	1,355 (95.2)	1.2 (0.8–1.9)
HES quintiles						
Lowest	346 (30.4)	794 (69.6)	0.9 (0.7–1.0)	71 (11.0)	574 (89.0)	**2.1 (1.4–3.0)**
Second	472 (32.0)	1,002 (68.0)	0.9 (0.7–1.0)	100 (12.5)	697 (87.5)	**2.2 (1.5–3.1)**
Third	617 (37.9)	1,011 (62.1)	1.1 (0.9–1.3)	58 (6.9)	779 (93.1)	1.1 (0.8–1.7)
Fourth	561 (36.9)	960 (63.1)	1.0 (0.9–1.2)	54 (6.5)	773 (93.5)	1.1 (0.7–1.6)
Highest	416 (36.2)	732 (63.8)	1	55 (6.8)	751 (93.2)	1
Marital status						
Married	2,186 (38.1)	3,559 (61.9)	**1.6 (1.4–1.9)**	260 (8.9)	2,663 (91.1)	0.8 (0.6–1.1)
Single	241 (20.1)	957 (79.9)	1	78 (7.8)	920 (92.2)	1

*Values in bold are significant (p<0.05). OR, odds ratio; HES, household economic status. OR was adjusted for sex and age.  †Includes construction, factory work, casual manual work on call, handicraft work, and mining. ‡Includes children, housewives, elderly persons, and retired persons.

**Table 2:** Variables in models predicting consumption of raw pig blood , Vietnam*

Group	Variables in final model†	Nagelkerke R‡
Rural persons	Sex, age, occupation, marital status, HES	0.123
Rural farmers	Sex, age, marital status, HES	0.086
Rural non-farmers	Sex age, occupation, marital status	0.168
Urban persons	Sex, age, occupation, HES	0.185
Rural and urban persons	Sex, age, occupation, marital status, location (rural vs. urban)	0.242

*HES, household economic status. †For model selection, all variables were forced into logistic regression. Each variable that was not significant (p ≥0.10) was removed step by step until all remaining variables were significant (p<0.10) in the model.  ‡Higher values indicate a stronger model.

More men than women reported consumption, and this difference was greater in the urban setting than the rural setting. Given that more women than men participated in the survey, the estimated frequency of persons consuming *tiet canh* will likely be higher than reported in this study. The practice was more common in persons 40–49 years of age than in other groups. Persons who reported highest consumption included farmers, manual laborers, persons working in service and sales. In the urban district, household economic status was negatively associated with consumption levels (odds ratio >2.0 for 2 lowest quintiles compared with the highest quintile). This finding was further confirmed in focus groups because *tiet canh* is relatively inexpensive and available in most markets. Therefore low-income workers are more likely to eat this dish (Technical Appendix Table 5).

## Conclusions

Consumption of *tiet canh* is closely linked with traditional family celebrations, particularly weddings. These traditions are a source of pride and social bonding among community members. Pigs are frequently slaughtered at homes of families hosting celebrations. Several male participants expressed pride and fond memories of their experience in participating in slaughtering events. *Tiet canh* is sometimes served at family celebrations expressly to demonstrate that slaughtered pigs are healthy. Cultural contributions of *tiet canh* must be understood to develop effective communication messages to reduce health risks associated with this practice.

Participants articulated strong confidence in the safety of raw pig products when the source of the pig was known to the consumer and the pig appeared healthy. Sources of pigs considered relatively safe were home-raised pigs, wild boars, or pig breeds locally known as ‘*lợn mán* and *lợn mường* (typically free-range, scavenging pigs raised by ethnic minorities). These perceptions contrast with findings of prevalence studies that showed high carriage rates of *S. suis,* even in apparently healthy pigs and pig products (*13*), and with reports of transmission of neurocystercercosis (*14*) and trichinellosis (*6**,**15*), which suggested increased transmission risks associated with scavenging pigs.

Beliefs about potential health benefits of eating *tiet canh*, such as preventing anemia or a general cooling effect, were widespread. However, participants did not fully understand the health risks posed by infectious agents or contaminants, and risks were dismissed or overlooked. Although concerns regarding the risk for diseases associated with *tiet canh* were raised in all focus groups, few participants knew what specific diseases are transmissible to humans through *tiet canh* consumption. In contrast, risk underestimation through optimistic bias was common, and fatalistic attitudes were shared in the group setting (online Technical Appendix).

The Agriculture Ministry of Vietnam had issued an official letter (no. 18 BNN/CĐ, May 21, 2009) that requested coordinated actions in controlling transportation, slaughtering, selling, and consumption of animals and animal products in response to recent disease reemergence. This letter also recommended a ban on selling of *tiet canh*. However, this proposed ban was considered to be unenforceable and ineffective among participants in all focus groups. The profit from selling *tiet canh* and consumer demand were considered key features that will perpetuate this traditional dish. Furthermore, trade in raw pig products is too widespread and decentralized, and the food chain from pig producers to pork consumers is too complex to enable regulation or enforcement of trade bans.

This study showed that consumption of *tiet canh* was more common among adult working-age men, outdoor workers, low-income urban inhabitants, and married persons in rural areas. Children rarely eat *tiet canh*, which may partly explain why *S. suis* meningitis is mainly a disease of adults and more common in men. Disease surveillance and reporting should be improved to better estimate the incidence of *S. suis* infections and clarify the relative role of the foodborne transmission route.

Given the traditions of consumption of *tiet canh* during family celebrations, interventions such bans on consumption or simple education messages on health risks without accounting for associated cultural values are unlikely to be effective. However, changes in education, urbanization, and increasing income levels will affect social and behavioral attitudes toward consumption of *tiet canh* in the future. Food safety research could benefit consumers by exploring methods of preparation of *tiet canh* designed to reduce infectivity of any pathogens in raw blood and preserve desired texture or taste characteristics of this traditional cuisine.

Technical Appendix Details on data collection, characteristics of participants, questionnaire, and data analysis for raw pig blood consumption and potential risk for *Streptococcus suis* infection, Vietnam.
