# Supplementary material for: Raw Pig Blood Consumption and Potential Risk for Streptococcus suis Infection, Vietnam
Source: Emerg Infect Dis. 2014 Nov;20(11):1895–8. doi: 10.3201/eid2011.140915 (PMC4214319; doi:10.3201/eid2011.140915)
Supplement: Technical Appendix — Details on data collection, characteristics of participants, questionnaire, and data analysis for raw pig blood consumption and potential risk for Streptococcus suis infection, Vietnam. [file 14-0915-Techapp-s1.pdf]

Article DOI: http://dx.doi.org/10.3201/eid2011.140915

Raw Pig Blood Consumption and Potential Risk for *Streptococcus suis* Infection, Vietnam

Technical Appendix

Methods

The 2 surveillance sites in Ba Vi and Dong Da, Vietnam, share common characteristics of typical rural and urban areas in northern Vietnam, and provide a good contrast between the 2 types of community settings in a rapidly urbanizing country. Each site contains ≈11,000 households, representing 20% and 12% of the district population in the Ba Vi and Dong Da districts, respectively.

Data Collection

We conducted a community survey about consumption of raw pig blood during May–June 2012 in Ba Vi and during December 2012–January 2013 in Dong Da. Trained interviewers visited the households as scheduled in their routine surveillance round and interviewed 1 household member about individual practices of consuming raw pig blood. Respondents were asked the following closed ended question for the food practice: "How many times have you eaten raw animal blood in the past year?" and answered by choosing one of the options offered: none, 1–3 times, 4–8 times, 9–12 times, >12 times, and unsure. Participants were required to specify the type of animal blood that they consumed, which include pig blood, duck blood, and other types of blood. All complete data forms were checked by field supervisors and a data clerk before data entry. Sociodemographic data, including sex, age, highest education level, marital status, occupation, and household economic status, of the respondents were extracted from the latest household update survey in both sites.

The follow-up qualitative study used a grounded-theory embedded design to uncover the experiences of participants related to raw pig blood eating and build the concepts from the data to explain the behavior. This study was conducted during April–June 2013 with 5 focus groups (FGs) in Bavi, including FG1–4 at participants' house and FG5 at the meeting room

of the FilaBavi office located in the district hospital, and 5 (FG5–10) in Dong Da, which were organized at the community health centers in the participants' vicinity. All FGs were moderated by one of the authors (VTLH) in Vietnamese by using a question guide. The question guide was designed to include 5 categories of questions to maintain the flow of the discussion: opening, introductory, transition, key, and ending (*1*).

The main aspects discussed are contexts in which the dish is consumed, attributes of the dish (likes and dislikes), factors influencing the choices of eating, perceived health benefits and health risks associated with the dish, feasibility of relevant public interventions, and recommendations for better implementation. Participants were invited to share their experiences of related public interventions. The subsequent discussion focused 4 main types of interventions: 1) information, education and communication activities, 2) the ban on selling raw animal blood, 3) inspection of animals and animal products, and 4) vaccination of pigs. The question guide was adjusted after each FG when there was new information identified. Each FG lasted 1.5–2 hours and was audiotaped with consent from the participants. One research assistant was present in each FG for note taking and logistics support. At the end of each FG, the moderator summarized the content discussed and checked with the participants if the moderator has correctly understood the participants' responses.

Eighty-one persons were purposefully selected from the 2 surveillance sites to participate in 10 FGs. These participants were met and invited individually by the field interviewers. In each site, we held 2 FGs for men and 2 FGs for women who were the general community residents, and 1 FG for local staff (health workers, women's union staff, and culture and information staff). Each FG had 6–9 participants. All FGs included persons who never consumed, who used to consume, and who were consuming raw pig blood dishes at the time of the study. The age range of the persons was 21–80 years (median age 45 years). Each FG participant was compensated ≈$9.50 (US dollars) for their time participating in the study.

Data Analysis

Quantitative data were analyzed by using SPSS software version 19.0 (IBM Corp., Armonk, NY, USA). We calculated the proportions of respondents eating *tiet canh* with 95% CIs. Associations with sociodemographic variables were examined by using univariate logistic regression with gender and age adjustment. Independent variables included sex, age, education (years of schooling), occupation, household economic status (quintiles), and marital status. Odds ratios with 95% CIs were calculated for each variable. Model selection was performed by forcing all variables into a model and removing step-by-step the least

significant variable. We used the Nagelkerke R^2 statistic, which can range from 0 to 1, to select the most parsimonious model with the highest explanatory power and smallest number of variables.

We transcribed qualitative FG data and uploaded to the NVivo software version 10 (QSR International Pty. Ltd., Doncaster, Victoria, Australia) for cleaning and analysis. Transcripts were read in Vietnamese by 1 of the authors (VTLH) to identify initial categories. They were then reread in detail, and each quote examined to see if it fit within existing categories or if a new category was required. Toward the end of the process, all transcripts were reviewed again to group responses into broader categories. Results were verified by another author (TKT), and validity of the categories was reviewed again by all team members. Representative quotes from each category are provided in this Technical Appendix. The FG discussions reached a point of saturation (no new information was identified in the last FG), and generated highly valuable observations that greatly informed all aspects of the research topic.

Ethical Considerations

The study was approved by the Oxford Tropical Research Ethics Committee of University of Oxford (OXTREC Reference 509–13) and the Hanoi Medical University Review Board. Household participants were part of the demographic surveillance cohorts for which informed consent was obtained.

Reference

Krueger RA, Casey MA. Focus groups: a practical guide for applied research. 3rd ed. Thousand Oaks (CA): Sage Publications; 2000.

Technical Appendix Table 1. Questionnaire regarding raw pig blood consumption and potential risk for *Streptococcus suis* Infection, Vietnam

Type of question	Content
Opening (5 min)	*For people who consume tiết canh*: Tell us who you are, what is you occupation, and when was the last time you eat tiết canh.<br>*For people who do not consume tiết canh:* Tell us who you are, what you do, and when was the last time you eat tiết canh, if ever.
Introduction (10 min)	What is the first thing that comes to mind when you hear the word “tiết canh”? What are the main ingredients for a plate/ bowl of tiết canh? Where do the main ingredients come from? Describe the process of making pig *tiet canh*.<br>*For people who consume tiết canh*: Think back to your last experience related to pig tiết canh, could you describe that experience (where, where, with whom, how were your feelings)?<br>*For people who do not consume tiết canh:* Think back to your last experience related to pig tiết canh (even when you did not eat it), could you describe that experience (where, with whom, your feelings)?<br>*For women who do not eat tiết canh:* Does your husband eat pig tiết canh? How do you feel about that?
Transition (15 min)	Who often eat tiết canh, what type of tiết canh, with what food/ drink and when/ where do they often eat it (*context*)?

Type of question	Content
	Context might include breakfasts, special traditional occasions (wedding, funeral, annual commemoration of death…), friend/ family gathering…<br>*Explore further if these traditional occasions/ friend or family gatherings are mentioned:* What attributes of tiết canh have made it a common food to serve at these events?
Key<br>Total:<br>75 min	*1. Attributes of tiết canh (10 min)*<br>What do you like about pig tiết canh?<br>What do you dislike about pig tiết canh?<br>*2. Influences to consume tiết canh (15 min)*<br>*For people who consume tiết canh*: Please tell us the three most important factors that often make you decide to eat pig tiết canh.<br>*For people who do not consume tiết canh*: Please tell us the three most important factors that often make you avoid eating pig tiết canh.<br>*Moderator/ assistant write all factors mentioned in the flipchart*<br>Now, look at the list of all these factors, which factor is the most important for you (not necessary the one that you have told us before)?<br>*3. Health risks associated with tiết canh (30 min)*<br>What are the good things to your health from eating pig tiết canh?<br>What are the bad things to your health from eating pig tiết canh?<br>Should children eat tiết canh? What are the reasons for children eat/ not to eat pig tiết canh?<br>Should women eat pig tiết canh? What are the reasons for women eat/ not to eat pig tiết canh?<br>*Show a picture of a patients with severe Streptococcus suis infection with skin necrosis.*<br>Look at this picture, what do you think has happened to this patient? And how?<br>*Explain what happened to the patient: Streptococcus suis infection after eating pig tiết canh. People can get this disease not only through eating tiết canh, but also through eating other undercooked pig products.*<br>*For people who consume tiết canh*: Knowing this possible consequence of eating pig tiết canh, will you still eat it, and what are the reasons for your choice?<br>*For all participants*: Pig organs such as uterus, intestines, tonsils or tongue are also the foods that can contain *S.suis* bacteria, and these dishes are often served undercooked. What are the reasons for these foods to be undercooked? From your experience, is this easy to prepare these foods well enough to kill harmful bacteria?<br>*4. Recommendations for interventions (20 min)*<br>Should we do something to prevent *S.suis* infection through eating pig tiết canh and other undercooked pig products? If yes, what should be done and how? (by individual, by government)<br>*Moderator list all suggestions from participants on the flipchart.*<br>Following is a list of possible programs that might be carried out to prevent *S.suis* infection through eating pig tiết canh and other undercooked pig products, please choose the most effective intervention that you think can work for your community. Provide reasons for your choice.<br>*Moderator shows the following list of interventions on a flipchart to the participants, and add any new interventions mentioned by the participants in the previous question:*<br>Public education campaign (via TV, radio, newspaper, loudspeakers…) to advice the public not to prepare or eat raw/ undercooked pig derived dishes<br>Public education programs to provide information for communities of how to prepare safer pig dishes from the high-risk pig products.<br>Public ban on selling tiết canh products and implementation of this ban<br>Vaccination of pigs against *S.suis*<br>Education at school on the health risks of eating raw/ undercooked pig products<br>Prohibition of selling, processing and consumption of sick/ dead pigs
Ending (10 min)	*Moderator summarizes the main content of the focus group.*<br>Is there anything else you would like to add or mention before we wrap- up?

Technical Appendix Table 2. Recipe commonly used for making *tiet canh* in northern Vietnam

Preparing blood: Pig is bled and blood is collected into a large container which already contains a small amount of saline solution to initially prevent the blood from coagulating. The saline solution is usually made from salt and water (or diluted fish sauce). If too much salt, the blood will not coagulate when mixing with the solid mix at the later stage.
Preparing the solid mix: varies depending on type of pig and geographic regions. Common pig portions used for making the solid mix are: spareribs meat, rib cartilage, neck meat, lung, tongue, throat, intestines. These are boiled/ roasted and chopped and maybe fried until crunchy. Roasted onion and ginger also can be added .Put the mix into bowls/ plates that will be used for preparing the final dish.
Add the prepared blood together with some water (suggested amount 1 spoon of blood and 2 spoons of water) to the bowl/ plate containing the solid mix above. Let it set until it coagulates.
When it coagulates, sprinkle some peppers and roasted peanuts on top.
Served with lemon, some aroma leaves such as mint leaves, basil, thorny coriander, and rice wine. Boiled intestines, stomach, and uterus can also be served with *tiet canh*.<br>(summarized from the transcripts of focus group discussions)

Technical Appendix Table 3. Demographic and economic characteristics and *tiet canh* consumption practice of respondents in the survey in Ba Vi (rural district) and Dong Da (urban district), Hanoi, Vietnam*

Characteristic	Ba Vi, n = 6,943	Dong Da, n = 3,921
Male sex	1,710 (24.6)	1,353 (34.5)
Age, y mean (95% CI)†	47.0 (46.6–47.3)	48.3 (47.8–48.8)
Education (no. schooling years)	7 missing	26 missing
≤5	1,110 (16.0)	148 (3.8)
6–9	4,310 (62.1)	852 (21.9)
10–12	1,351 (19.5)	1,574 (40.4)
>12	165 (2.4)	1,321 (33.9)
Occupation	28 missing	22 missing
Office worker	168 (2.4)	1,155 (29.6)
Manual laborer‡	666 (9.6)	339 (8.7)
Farmer	4,474 (64.7)	2 (0.1)
Services and sales	561 (8.1)	923 (23.7)
Other	260 (3.8)	56 (1.4)
Not in the workforce§	786 (11.4)	1,424 (36.5)
HES (quintiles)	32 missing	9 missing
Lowest	1,140 (16.5)	654 (16.5)
Second	1,474 (21.3)	797 (20.4)
Third	1,628 (23.6)	837 (21.4)
Fourth	1,521 (22.0)	827 (21.1)
Highest	1,148 (16.6)	806 (20.6)
Marital status		
Married	5,745 (82.7)	2,923 (74.5)
Single	1,198 (17.3)	998 (25.5)
Consumed *tiet canh* in the past year		
No. of times	4,516 (65.0)	3,583 (91.4)
1–3	1,538 (22.2)	240 (6.1)
4–8	628 (9.0)	56 (1.4)
9–12	158 (2.3)	17 (0.4)
>12	103 (1.5)	25 (0.6)

*Values are no. (%) unless otherwise indicated. HES, household economic status
†Age range is similar between two sites: 8–97 in Ba Vi and 9–102 in Dong Da.
‡Includes construction, factory work, casual manual work on call, handicraft work, and mining.
§Includes children, housewives, elderly persons, and retired persons.

Technical Appendix Table 4. Proportions reporting eating *tiet canh* by type of animal blood, Vietnam

Type of animal	Ba Vi, no. (%)	Dong Da, no. (%)
Pig	2427 (35.0)	338 (8.6)
Duck	557 (8.0)	324 (8.3)
Other animal (goat, cow, dog, horse, rabbit)	10(0.14)	19(0.48)

Technical Appendix Table 5. Characteristics of 81 participants in 10 focus group discussions, Vietnam*

Characteristic	Ba Vi, n = 40	Dong Da, n = 41
Male sex	18 (45)	16 (39)
Mean age, y (95% CI)	39.9 (37.1–42.8)	49.6 (45.1–54.2)
Type of participants		
General community	34	33
Health workers	4	3
Other community workers	2	5
Consumed raw animal blood	26 (65.0)	26 (63.4)
Type of blood often consumed		
Pig	17 (65.4)	2 (7.7)
Duck	2 (7.7)	17 (65.4)
Pig and duck	6 (23.1)	4 (15.4)
Where *tiet canh* usually consumed		
Home	17 (65.4)	10 (38.5)
Friend’s house	6 (23.1)	4 (15.4)
Shop	2 (7.7)	9 (34.6)
Consume pig organs	38 (95.0)	37 (90.2)
Consumed undercooked pig organs	2 (5.3)	8 (21.6)
Intention to change *tiet canh* eating behavior†		
Change behavior (stop or reduce)	9 (34.6)	13 (50.0)
Still eating	10 (38.5)	8 (30.8)
Unsure/ no response	7 (26.9)	5 (19.2)

*Values are no. (%) unless otherwise indicated.
†After discussing about the picture of patient with *Streptococcus suis* infection.

Technical Appendix Table 6. Factors influencing participants' choice of eating *tiet canh* explored from focus groups discussions, Vietnam*

Category	Descriptions
Food characteristics	Taste, appealing appearance, easy-to-eat
Social contexts	Weddings (in rural area), parties, slaughtering events: *Tiet canh* eating was most commonly learned in social occasions such as rural weddings, New Year celebrations and other special gatherings. Pigs are often slaughtered in the early morning, *tiet canh* is made and consumed as soon as possible within the morning when it is still fresh. Weddings were the most frequently reported occasion for *tiet canh* consumption in the rural area. Indeed, among young rural women, their first reported instance of *tiet canh*<br>Drinking encounters (best matched food for alcohol drinking) at home or in shops: Many shops sell fresh *tiet canh* in the morning, however it is also sold for lunch and dinner.<br>Family contexts: children copy adults within the family to eat *tiet canh,* parents made it as a special treat for children, encouraged children to eat due to belief that it provides improved immunity
Availability and accessibility	Available in social parties;<br>Available in shops at affordable price: *Tiet canh* can also be found in most markets in both urban and rural settings, and is relatively cheap (≈10–20,000Vietnam Dong, equivalent to <$US 0.5–1).<br>High profit made from selling the dish;
Perceived benefits	Mentioned by 44% (n = 36) of respondents<br>The most commonly mentioned benefit was the notion that *tiet canh* is a 'cold' food that serves to ease internal body heat or could be helpful in treating mouth ulcers (n = 24, mainly in urban FGs).<br>Other perceived benefits included preventing anemia, treating headache, cough and dysentery, detoxification, clean the body internal system, or clearing minute dust in the lungs (e.g., hair clippings).<br>Myth: *Tiet canh* was considered a 'red' food associated with good luck if eating *tiet canh* on the 1st day of the lunar month (n = 13, mainly in urban FGs).
Perceived safety	Safe food source: Home grown pig/ self-made dish, free-range pig, rural pig, wild boar, trustworthy/ known source; Perceived *tiet canh* was safe when the source of the pig or the person preparing the dish was known to the consumer (mentioned by 61% and 50% of rural and urban participants, respectively). Even when the blood was bought at the wet market, people considered *tiet canh* to be more safe when prepared by someone they knew and trusted, versus an unknown commercial source.<br>The word 'clean' was used by a substantial number of consumers (31%, n = 16) to describe deliberate choices on whether to eat *tiet canh* in a given context, and it conveyed two different meanings: the source was known or was perceived as prepared under trusted hygienic conditions<br>Drinking alcohol: Several men expressed the belief that rice wine can help mitigate possible health risks by "disinfecting" *tiet canh*. This type of drink is regularly served to pair with *tiet canh*. In addition, alcohol helps reduce the bloody smell of the dish, and neutralize the dish (hot and cold food).
Disease risk awareness	Awareness that the dish can transmit diseases to humans;<br>Pig outbreaks (such as porcine respiratory and reproductive syndrome [PRRS] and foot and mouth diseases): The presence of ongoing disease outbreaks in pigs negatively influenced consumption patterns, and discouraged some people from eating. This effect was noted more frequently among rural participants, and in communities with a history of pig outbreaks (FG1,2,4)<br>Risk underestimation through optimistic bias: attitudes such as diseases can't happen because I am resistant (FG3,6,7,8), diseases happen somewhere else but not here (FG6).<br>Confidence in the relative safety of *tiet canh* was expressed by many participants based on repeated personal experience: I've never had any bad experience after eating *tiet canh* (FG1,4,7,8).<br>Fatalistic attitude: death is our fate.<br>Some participants, despite being aware of health risks and having stopped consumption for some time, resumed eating *tiet canh* again in situations where they believed the risk was low (e.g., a woman in FG10 ate when she was given *tiet canh* prepared from a lợn Mán).

*FG, focus group.

Technical Appendix Table 7. Key perceptions about public health interventions, Vietnam*

Type of intervention	Key perceptions
Information, Education, Communication activities	Mass media was not perceived as an effective method to reach certain populations such as mobile manual workers, men, heavy drinkers and older generations (FG1,9,10).<br>Communication messages should be repeated frequently and sustained over time to be most effective (FG8, 10).

Type of intervention	Key perceptions
Pig vaccination against *S. suis*	Pig vaccination overall was perceived as an effective measure, but most farmers were not aware of *S. suis* bacteria in pigs, probably due to the unavailability of an effective vaccine for this disease in the local market and thus lack of information on this specific pathogen.<br>Some concerns were expressed about current government programs for animal vaccination, including possible disease spread due poor biosecurity practices of field teams responsible for vaccination, insufficient vaccine supply and limited coverage (FG1, 4).
Ban on selling *tiet canh*	Considered to be infeasible and ineffective among participants in all FGs.<br>Monetary profit from selling *tiet canh* and the consumer demand were considered as key features that will perpetuate this traditional dish.<br>Trade in raw pig products is too widespread and decentralized, and the food chain from pig producers to pork consumers too complex to enable regulation or enforcement of trade bans.
Food inspection	Not confident in the standards of food inspection programs<br>Complained that sellers of unsafe or contaminated products are able to bypass inspections and they do not trust certified food

*FG, focus group.

Technical Appendix Table 8. Illustrative quotes for each category taken from the transcripts of 10 focus group discussions in Ba Vi and Dong Da, Hanoi, Vietnam*

Factor	Representative quotes
Food factor	*“I feel it is very delicious and cool. It is more special than other dishes...”* (F, age 43, rural, FG3)<br>*“Tiet canh is a favorite food. Why? Putting a piece of tiet canh into the mouth there is a buttery taste, not much bloody taste, crunchy, second it feels cool. There is that feeling in the mouth, and with some herbs, some lemon, some chilly, it feels sour and hot...”* (M, age 63, urban, FG6).<br>*“The main ingredient is pig raw pig blood. Also include pig cartilages which is chopped, roasted onion, mint leaves,...”* (M, age 41, rural, FG2).<br>*“Good food is often ordered for parties including pig tiet canh which contains throat meat, liver, and lung... These are boiled, roasted, chopped and fried, then added to tiet canh bowl so it will be very tasty, crunchy and has good flavor.”* (F, age 49, rural, FG3).
Social contexts	*“When I got married I ate tiet canh. I followed my husband to come here, the elderly slaughtered pigs and made tiet canh... then I got familiarized with the dish.”* (F, age 29, rural, FG3).<br>*“My children eat it every Saturday and Sunday... if they have enough time in the morning they will eat it for breakfast’*” (F, age 33, rural, FG3).<br>*“Generally going out to eat chao long tiet canh* (soup with pig intestines and raw pig blood dish) *has to be in a group of two to three people so you feel fun and the dish is delicious, otherwise I don’t wanna go’*” (F, age 48, urban, FG9).<br>*“...we often eat it together, gather together, they eat so I also eat..’*” (M, age 46, rural, FG5).<br>*“I think I do not really like it, just sit together and eat together for fun, eat a little not much and not frequently”* (F, age 29, rural, FG3).<br>*“At the time near Tet* (Lunar new year celebration) *... we made tiet canh. Three to four households for example shared one pig (for slaughtering) and then we had to prepare food for the men sitting together, on bowl tiet canh each and rice wine”* (F, 40, urban, FG9).<br>*“Most people eat it, little or a lot, never say I never eat it. Thirty days in a month, twenty-seven days with weddings already, how can I avoid tiet canh?”* (M, age 45, rural, FG4).
Source of food	*“I will eat provided that I know its source, whether it is home grown or from a clean source and second it was made carefully, and cleanly by the family then I will eat two bowls, not one.*” (M, age 63, urban, FG6).<br>*“I went to the highlands, pig from the forest, slaughtered and the tiet canh was yummy, I still crave for it...”* (F, age 56, urban, FG10).

Factor	Representative quotes
Disease risk awareness	*"The thing I don't like about tiet canh is during outbreaks so I don't eat. And hearing from the mass media, radio, newspaper about the outbreaks, all types of outbreaks such as flu, then I never eat... until things are normal. When the news announce that outbreaks are over, we start to eat again"* (M, age 41, rural, FG2).<br>*"Death is our fate, now restaurants here do not make tiet canh for us to eat, only in town they still make it, not here, for a long time already"* (M, age 45, rural, FG4).<br>*"If there are outbreaks, we only eat tiet canh when knowing where the pig comes from, if no outbreaks we go to restaurants to eat a bowl of tiet canh everyday"* (M, age 45, rural, FG4).<br>*"Even when they heard about it* (disease outbreak in pigs), *nothing changes. Obviously hand-foot-mouth and blue ear pig outbreaks in the village, they still continue shared slaughtering. Last year, Mrs. N, the blue ear outbreak was in the village, (pigs) died in nearly all households, but in the last household where pigs were not yet diseased they still shared to slaughter a pig of 70–80kg, 7–8 households*' (F, age 47, rural, FG1).<br>*'I like it because it is tasty, but thinking of many outbreaks and diseases make me scared... I stop eating because of too many disease outbreaks."* (F, age 50, urban, FG10).<br>*"Eating tiet canh will be good for your blood, second it has the cooling effect, but now there are too many diseases so we need to be alert, reduce it, should not eat."* (M, age 63, urban, FG6).<br>*"The mass media report that tiet canh has many diseases so I avoid tiet canh"* (M, age 22, rural, FG2).<br>*"First it is unhygienic. Second the problem that they* (the sellers) *want to get more profit so they make it from rubbish ingredients, no conscience, so we are afraid."* (F, age 41, urban, FG7).<br>*"Migrant workers, low awareness, don't care anything, neither their health... they still eat."* (F, age 48, urban, FG10)<br>*"I see that nothing bad happens* (after eating tiet canh)*... They can eat it then I can also eat it. There is nothing happening after eating, not die then it is OK."* (F, age 47, rural, FG1)
Perceived benefits	*"I often have internal body heat, regularly eating fruits but no effect. It is too unbearable when you have internal body heat. Eating tiet canh you feel it is eased right away..'"* (F, age 56, urban, FG10).<br>*"These days, in the north, on the 1st day of the lunar month, staff go to that restaurant to eat either steamed or raw tiet canh for good luck in their jobs... it has become a trend. My niece who opens a restaurant there can sell 500 bowls in a morning..."* (M, age 65, urban, FG6).
Drinking alcohol	*"Nobody eats tiet canh by itself, at least you've got to have a cup of rice wine."* (M, age 41, rural, FG2).<br>*"Frequent eaters are those who can drink rice wine, those who can only have sugary drinks* (with tiet canh) *will probably get diarrhea."* (M, age 22, rural, FG2).<br>*"I think bacteria will die when there is rice wine."* (M, village health worker, age 41, rural, FG5).
Availability/ accessibility	*"Wake up in the morning, they already sit there at tables on the roads, in the market in B20."* (F, age 69, urban, FG7).<br>*"Not only it is men's favorite, but selling it make a lot of money, high profit so in any circumstances they will sell it." (F, age 47, rural, FG1).*<br>*"If eating out, tiet canh is the most suitable food for our pocket money."* (M, age 45, rural, FG4).<br>*"...laborers often eat tiet canh more. In my opinion, it's simply because it is low-price."* (M, age 48, urban, FG8).
Information, education, communication	*"Communication campaigns can only reach more perceptive people, especially the mass media... The remaining groups including mobile manual laborers, low awareness, don't care anything... they still eat."* (F, age 48, urban, FG10).<br>*"A while ago TV mentioned about eating tiet canh and Streptococcus suis infection, at that time I stopped eating. Then when it no longer talked about it, I thought the outbreak was over... so TV, radio and newspapers should continue talking about this, eating tiet canh will decrease."* (F, age 33, rural, FG3).<br>*"I think communication campaigns should be longer. Not one period with one or two days, need to repeat, one week, ten days or more, if someone could not hear it today they can hear it at another time."* (M, age 48, urban, FG8).
Food inspection	*"To prevent* (infections), *first, the commune units for animal products inspection need to continue to inspect the hygiene and safety of food products regularly."* (M, age 63, urban, FG6).<br>*"First inspection, have you done it right? ...We don't know if there is an outbreak, we sell, the pig blood looks still clean, so we still eat. Who will check if this pig has enough qualities for sale or it is diseased and therefore not allowed for selling?"* (M, age 45, rural, FG4)<br>*"In the early mornings the inspector goes to check* (the meat products in the market), (the seller) *gives him money then he will give a stamp* (on the product), *he doesn't care what is what* (the quality)*..."* (F, age 29, urban, FG10).<br>*"About inspection, in reality they* (the sellers/ transporters) *still transport many, one truck with hundreds of pigs, then to the market, clearly there are stamps on the pork but do they really know what the quality is, stamp then collect money... we still need to go to the market, don't know which pig is good, which one is sick."* (F, age 48, urban, FG9).
Ban on selling raw animal blood	*"The trade is everywhere at small scales in this rural area, can't control. Even when they come, they did not have strong actions on the people they know."* (F, age 38, rural, FG1).<br>*"Ban it* (*tiet canh) but they* (the sellers) *still sell it illegally... They keep it in the fridge inside, anyone who wants to eat can ask and they will bring it out."* (F, age 50, urban, FG10).<br>*"There is still a demand, then no one able to control, can you control it both day and night? So you can prohibit them now, they continue it another time, you punish them with big money they still keep the practice."* (M, age 63, urban, FG6).

Factor	Representative quotes
Pig vaccination	*"All farmers who breed pigs vaccinate. Even in the small households, if one or two pigs they won't, but if three four or more pigs they vaccinate. Not much for Streptococcus suis vaccine, but other vaccines..."* (M, age 54, rural, FG2).<br>*"Vaccination is not a guarantee, there was one staff going from door to door with just only one syringe, not safe..."* (F, age 48, rural, FG1).<br>*"My household has 30 pigs, they gave us just one dose for one pig, they told me to inject the pigs myself... Drugs were provided abundantly somewhere else but not here..."* (M, age 45, rural, FG4).<br>*"I think to maintain the shops selling tiet canh and pork intestines porridge, the most important thing is to ensure the good quality products from the breeders and pig farming. Second they need to vaccinate the pigs carefully. I think in this case, it is OK to eat tiet canh."* (M, middle age, urban, FG8).

*FG, focus group.
